# Acute oxalate nephropathy: A potential cause of acute kidney injury in diabetes mellitus—A case series from a single center

**DOI:** 10.3389/fmed.2022.929880

**Published:** 2022-08-26

**Authors:** Daorina Bao, Yu Wang, Xiaojuan Yu, Minghui Zhao

**Affiliations:** ^1^Renal Division, Department of Medicine, Peking University First Hospital, Institute of Nephrology, Peking University, Key Laboratory of Renal Disease, National Health and Family Planning Commission of the People’s Republic of China, Key Laboratory of Chronic Kidney Disease Prevention and Treatment, Ministry of Education, Beijing, China; ^2^Laboratory of Electron Microscopy, Pathological Centre, Peking University First Hospital, Beijing, China; ^3^Peking-Tsinghua Center for Life Sciences, Beijing, China

**Keywords:** oxalate nephropathy, diabetes mellitus, acute kidney injury, hyperoxaluria, inflammasome, prognosis

## Abstract

**Background:**

Acute oxalate nephropathy (AON) is an uncommon condition that causes acute kidney injury (AKI), characterized by the massive deposition of calcium oxalate crystals in the renal parenchyma. In previous studies, urinary oxalate excretion has been found to be increased in patients with diabetes mellitus (DM). Here, we report a case series of diabetic patients with AKI with biopsy-proven AON, aiming to alert physicians to the potential of AON as a trigger of AKI in diabetic patients in clinical practice.

**Materials and methods:**

Cases with pathological diagnosis of AON who presented with AKI clinically and had DM between January 2016 and December 2020 were retrospectively enrolled. Their clinical and pathological manifestations, treatment, and prognosis were collected.

**Results:**

Six male patients with biopsy-proven AON out of a total of 5,883 native kidney biopsies were identified, aged 58.3 ± 9.1 years at the time of kidney biopsy. Only one patient who had received Roux-en-Y gastric bypass surgery took oxalate-rich food before the onset of the disease. None of them had clinical features of enteric malabsorption. Three patients were currently on renin-angiotensin system inhibitor treatment for hypertension, and 5 of them received non-steroidal anti-inflammatory drugs. Three patients presented with oliguria and 4 patients needed dialysis at the beginning with none requiring dialysis at discharge. Four patients received a course of corticosteroid treatment empirically. Among them, two patients had estimated glomerular filtration rate (eGFR) recovered to over 60 ml/min/1.73 m^2^, while the other two patients remained with kidney dysfunction at the last follow-up. In two patients without corticosteroid treatment, one patient fully recovered with eGFR over 90 ml/min/1.73 m^2^ and the other patient remained with kidney dysfunction at the last follow-up.

**Conclusion:**

AON might be a rare but potentially trigger of AKI in patients with DM. A kidney biopsy could help physicians to make the correct diagnosis. The proper treatment to alleviate oxalate-induced injury needs to be further studied.

## Introduction

Patients with diabetes mellitus (DM) may develop diabetic kidney disease (DKD), which develops progressively with a gradual decline in kidney function. Therefore, causes other than DKD should be differentiated when acute kidney injury (AKI) occurs in patients with DM. Acute oxalate nephropathy (AON) is diagnosed by the characteristic finding of widespread deposition of oxalate crystals in the kidney, which is a relatively uncommon cause of AKI in clinical practice (1, 2). The kidney serves as the main excretory organ of circulating oxalate. Patients with DM have been found to have increased urinary oxalate excretion (3–5). Furthermore, the prevalence of DM as comorbidity is approximately 40% in the reported cases of AON in the literature (6).

Elevation of plasma oxalate could lead to hyperoxaluria, which might induce oxalate crystal deposition in the kidney under certain circumstances. The causes of hyperoxaluria can be either primary or secondary (4). Primary hyperoxaluria refers to a group of autosomal recessive disorders with enzymatic defects in the glyoxylate pathway, which results in overproduction of oxalate and subsequent hyperoxaluria (7). Recurrent urolithiasis is the common clinical presentation of primary hyperoxaluria, which generally progresses to end-stage kidney disease (ESKD). Secondary hyperoxaluria is more common than primary hyperoxaluria. Oxalate may be derived exogenously from increased dietary oxalate intake and/or net availability of oxalate in the intestine (8). However, some patients have no evidence of exogenous oxalate derivatives, indicating the potential role of endogenous synthesis in the development of secondary hyperoxaluria. Interestingly, some oxalate precursors have been found with increased circulating levels in patients with DM, which are also identified as potential metabolite markers of DM in recent studies (9, 10). Here, we report 6 cases of AKI with biopsy-proven AON in patients with DM who were admitted to Peking University First Hospital, aiming to alert physicians about the potential of AON as a trigger of AKI in patients with DM in clinical practice.

## Materials and methods

### Patients

This retrospective analysis enrolled 6 patients with biopsy-proven AON who underwent native kidney biopsy at Peking University First Hospital from January 2016 to December 2020. The inclusion criteria were as follows: (1) AKI (defined as an increase in serum creatinine to ≥1.5 times baseline, which is known to occur within the prior 7 days) or AKD (defined by > 50% increase in serum creatinine within 3 months) clinically; (2) extensive oxalate crystal deposition in the kidney associated with tubular injury, obstruction, interstitial inflammatory cell infiltration, and fibrosis; (3) exclusion of other causes of kidney disease apart from non-specific microvascular (nephrosclerosis) and diabetic nephropathy; and (4) DM. The study was approved by the ethics committee of Peking University First Hospital and performed in accordance with the Declaration of Helsinki. Informed consent was obtained from each patient at kidney biopsy.

### Clinical data

Demographic (age, sex), medical history (hypertension, diabetes, hyperuricemia/gout, chronic kidney disease, urolithiasis, and surgery history), biological data [body mass index (BMI), systolic blood pressure (SBP), and diastolic blood pressure (DBP)], and conditions known to predispose patients to hyperoxaluria were collected from electronic medical charts. Laboratory data of serum and urinalysis at the time of renal biopsy were also collected. Medication information, including renin-angiotensin system inhibitors (RASIs), diuretics, non-steroidal anti-inflammatory drugs (NSAIDs), oxalate precursors, and antibiotics, was recorded.

### Urine oxalate measurements

Frozen spot urine samples stored at –80°C were thawed overnight at 4°C. Then, the samples were centrifuged, and the supernatants were collected, acidified with hydrochloric acid, and further deproteinized by filtration through a Dionex OnGuard II RP 1 cc Cartridge (Thermo Fisher Scientific, United States) before measurement. Oxalate measurement was performed by Aquion Reagent-free Ion Chromatography (RFIC) (Thermo Fisher Scientific, United States).

### Definition

Diabetes mellitus was defined as fasting plasma glucose ≥7.0 mmol/l, a self-reported history of diabetes, or receiving either insulin or oral antidiabetic drugs. Hypertension was defined as blood pressure (BP) higher than 140/90 mm Hg and/or the current use of antihypertensive medications. eGFR was calculated by the Chronic Kidney Disease Epidemiology Collaboration (CKD-EPI) equation. Hematuria was defined as red blood cell (RBC) > 3/high-power field. Leukocyturia was defined as white blood cell (WBC) > 5/high-power field.

## Results

We identified 10 cases with pathological findings of extensive oxalate deposition out of a total of 5,883 native kidney biopsies performed between January 2016 and December 2020. Three patients were excluded for concurrent findings of other kidney diseases [one patient with crescentic immunoglobulin A (IgA) nephropathy, one patient with focal segmental glomerular sclerosis, and one patient with membranous nephropathy]. Another patient was further excluded for the absence of DM. Finally, six cases were included in the present study.

Table 1 displays the demographic, clinical features, and potential hyperoxaluria enabling factors of the patients with AON in the present study. In brief, all the patients were male, and aged 58.3 ± 9.1 years at the time of kidney biopsy. No patient had a previous history of kidney disease, except one patient with a history of urolithiasis. Three patients were on RASI treatment for hypertension comorbidity. Five patients were found to have concurrent NSAID use at disease onset. Only one patient reported taking spinach in usual amounts before the onset of the disease, who also received Roux-en-Y gastric bypass surgery for gastric carcinoma 1 year ago and had acute diarrhea at the onset of the disease. No one patient reported chronic clinical steatorrhea that indicated malabsorption.

**TABLE 1:** Demographic, clinical features and potential hyperoxaluria enabling factors of 6 patients with acute oxalate nephropathy.

Patients	1	2	3	4	5	6
Age at onset	50	74	53	57	51	64
Sex	Male	Male	Male	Male	Male	Male
BMI (kg/m^2^)	23.5	24.2	28.3	18.9	25.7	19
Blood pressure (mmHg)	120/80	165/83	190/94	135/74	132/80	129/79
HbA1c	8.70%	6.40%	NA	6.70%	5.70%	5.90%
**Medical history**						
Diabetes mellitus	Yes	Yes	Yes	Yes	Yes	Yes
Duration (years)	13	Unknown	10	10	4	10
Treatment (OAD/Insulin)	Insulin	OAD	OAD	Insulin	OAD	OAD
Hypertension	Yes	Yes	Yes	Yes	No	Yes
Duration (years)	13	50	0.5	NA	/	10
Treatment	RASI + β-Blocker	CCB	RASI + β-Blocker	CCB	/	RASI
Chronic kidney disease	No	No	No	No	No	No
Nephrolithiasis	No	Yes	No	No	No	No
Hyperuricemia/gout	No	No	Yes	No	No	No
Surgery history	No	No	No	No	No	Roux-en-Y bypass surgery
Diuretic use	No	No	No	No	No	No
NSAID use	Yes	Yes	Yes	Yes	No	Yes
**Hyperoxaluria-enabling factors**						
Increased intake of oxalate precursors	No	No	No	No	No	Spinach
Increased oxalate availability in the colon due to fat malabsorption						
Chronic pancreatitis/pancreatic insufficiency	No	No	No	No	No	No
Roux-en-Y bypass surgery	No	No	No	No	No	Yes
Decreased intestinal oxalate degradation						
Recent antibiotic use	No	No	No	No	No	No

NA, not available; OAD, oral antidiabetic drugs; NSAIDs, non-steroid anti-inflammatory drugs.

**FIGURE 1:**
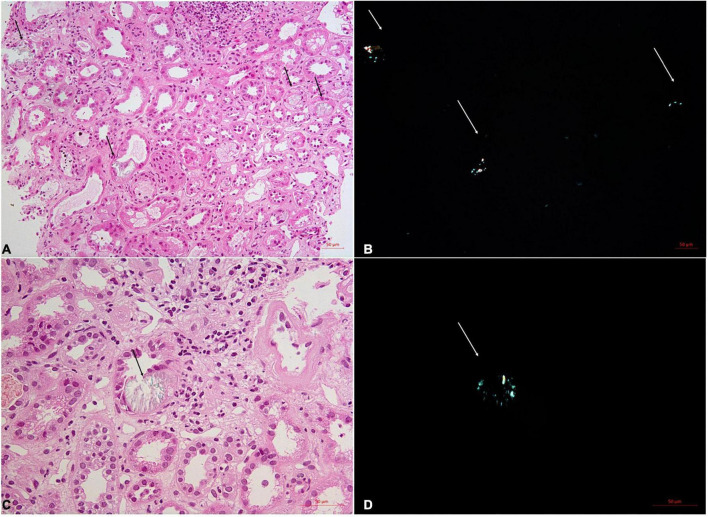
Renal biopsy of the No. 5 patients with acute oxalate nephropathy. On light microscopy, H&E staining shows acute tubular injuries with numerous intratubular oxalate crystals (**A,C**, arrows). These crystals demonstrate birefringence under polarized light (**B,D**, arrows). Original magnification 200X **(A,B)** and 400X **(C,D)**.

Regarding pathological findings, acute tubular epithelial injury with extensive oxalate deposition was the most prominent finding. The tubular cells presented with vacuolar and granular degeneration and effacement of the brush margin, with occasional atrophy found. The interstitium was edematous with the focal distribution of fibrosis. Focal infiltration of lymphocytes and monocytes was observed in all the specimens, with scattered eosinophil infiltration found in 4 cases. None of the cases were found with oxalate crystal deposition in the glomeruli. Instead, ischemic glomerular sclerosis and ischemic wrinkling of the glomerular basement membrane (GBM) to different degrees were observed in these cases. Some also had mild segmental mesangial cell proliferation and matrix accumulation, accompanied by a thickness of GBM observed under electron microscopy. Arteriosclerosis and hyalinization of arterioles were also observed. The typical pathological changes are shown in Figure 1. The polarized light microscopy image and periodic acid-Schiff (PAS) staining for each patient are shown in Supplementary Figure 1.

Table 2 shows the clinical presentation, treatment strategy, and disease course of the AON of the 6 patients. Three patients presented with oliguria at the onset of disease, and one patient reported approximately half daily urine output compared to usual, with peak creatinine ranging from 463 to 1,641 μmol/l. Neither leukocyturia nor urine crystals were found in the sediments of urine. Two patients without oliguria received hydration, and the other patients received renal replacement therapy, with none requiring dialysis at discharge. Alkali therapy with citrate was given to 4 patients. Four patients received a course of corticosteroid treatment, and two of them had eGFR recovery to over 60 ml/min/1.73 m^2^ at the last follow-up, while 2 patients remained with kidney dysfunction. Regarding the remaining 2 patients who did not receive corticosteroids, one patient experienced full recovery with eGFR over 90 ml/min/1.73 m^2^, while the other patient remained with kidney dysfunction at the last follow-up.

**TABLE 2:** Clinical presentation at admission, treatment strategy and disease course of the 6 patients with acute oxalate nephropathy.

Patients	1	2	3	4	5	6
**Renal status at presentation**						
Oligoanuria/anuria	No	No	No	Yes	Yes	Yes
Peak Serum creatinine (μmol/L)	463	684	1282	1461	900	471
eGFR (ml/min/1.73m^2^)	11.8	6.2	3.4	2.8	5.3	10.5
Calcemia (mmol/L)	2.62	2.04	2.3	2.05	2.17	2.39
Phosphatemia (mmol/L)	1.32	1.32	2.48	1.3	1.38	1.55
Urine PH	5	6	5	8	6.5	5
**Urine analysis**						
Leukocyturia	No	No	No	No	No	No
Hematuria	No	No	No	No	RBC 7-8/HPF	No
Crystals	No	No	No	No	No	No
ACR (mg/g)	12.43	67.63	107.27	95.77	58.76	15.35
Ca/Cr (mmol/mmol)	0.15	0.27	0.04	0.34	0.17	0.18
Oxalate/Cr (mg/g)	9.49	10.92	6.68	17.43	10.14	9.24
**Treatment strategy**						
Renal replacement therapy	No	No	HD	HD	HD	HD
Corticosteroid therapy	No	Yes	Yes	Yes	Yes	No
Dose of Prednisone	/	30mg	30mg	50mg	30mg	/
Hydration	Yes	Yes	No	No	No	Yes
Alkalinize urine	Citrates	Citrates	No	No	Citrates	Citrates
Pyridoxine	No	No	No	No	Yes	No
Calcium supplements	No	No	No	No	No	Yes
**Renal status at discharge**						
Length of stay (days)	13	37	13	40	20	16
Serum creatinine (μmol/L)	147	200	555	231	138	346
eGFR (ml/min/1.73m^2^)	47.0	27.5	9.3	26.0	50.6	15.2
**Renal Outcome at last follow-up**						
Duration of follow-up (months)	48	4.5	2.5	1.5	5	2.6
Serum creatinine (μmol/L)	85	146	108	175	100	200
eGFR (ml/min/1.73m^2^)	91.6	40.2	67.1	36.4	74.7	28.5

Ca/Cr, calcium creatinine ratio; Oxalate/Cr, oxalate creatinine ratio.

## Discussion

In the present study, we reported 6 cases of biopsy-proven AON that presented with AKI in patients with DM. No one was considered to have oxalate nephropathy before the biopsy. Here, we described their clinical manifestations, natural history, and prognosis, aiming to trigger physicians’ alert about AON as a potential cause of AKI in the setting of DM.

Kidney excretion accounts for the majority of daily oxalate excretion from the body. Circulating oxalate is freely filtered at the glomerulus, reabsorbed, and secreted by the proximal tubule (10). Multiple studies have shown that the 24-h urine excretion of oxalate was higher in individuals with DM than in those patients without DM (3, 4). Nephrolithiasis is a common comorbidity of DM, with calcium oxalate as the most common composition of stones (11). In a retrospective analysis of 462 patients with nephrolithiasis, patients with DM excreted greater urinary oxalate than those patients without DM (3). In a recent report from the Chronic Renal Insufficiency Cohort (CRIC) Study with 3,123 established CKD, individuals with DM had 11% higher 24-h urinary oxalate excretion than those patients without diabetes. DM was found to be independently associated with higher urinary oxalate excretion after adjustment for body mass index, age, race, sex, medications, and laboratory tests (5). These results indicate that patients with DM are at risk for hyperoxaluria-enabling conditions.

Hyperoxaluria can develop from multiple causes. Urinary oxalate is derived either exogenously from dietary oxalate intake and net intestinal absorption or endogenously from oxalate synthesis mainly in the liver (10). Most reported AON cases in the literature have attributed the cause to either high intakes of oxalate-containing foods or oxalate precursors (12–15) or malabsorption induced by short-bowel syndrome (16), including celiac disease (17), gastric surgery (Roux-en-Y gastric bypass) (18), inflammatory bowel disease (19), chronic pancreatitis or pancreatic insufficiency, *etc.* (20, 21). In the present study, all the patients were further extensively questioned about the dietary source of oxalate or oxalate precursors such as ascorbic acid, fruit juice, and ketogenic diet after AON was confirmed pathologically. Only the patient who had a history of Roux-en-Y gastric bypass surgery reported taking spinach. Although diabetic patients may have undetected pancreatic exocrine insufficiency (22), no patients had clinical features of malabsorption in the present study. These 6 patients were all the diabetic patients. Glyoxylate is an immediate precursor of oxalate. In recent years, glyoxylate has been identified as a potential metabolite marker of type 2 DM (10). A retrospective study showed that the plasma level of glyoxylate was elevated in diabetic subjects, even up to 3 years before diabetes diagnosis (9). Glyoxal, another important precursor involved in endogenous oxalate synthesis in humans, has also been found to have higher plasma levels in diabetic patients than in controls, as well as increased urinary oxalate excretion (23). Therefore, the potential contribution of these metabolic components of diabetes to the development of hyperoxaluria in DM needs to be studied more.

In the present study, all of our patients presented with AKI with the finding of universal oxalate crystal deposition in tubules and/or the interstitia in kidney biopsy. The deposition of oxalate in the kidney is subjected to several factors, including supersaturation, precipitation, crystal aggregation, and adhesion (1). Decreased urine output with severe supersaturation may cause massive crystal deposition, renal epithelial cell damage, inflammation, and necrosis, resulting in AKI (24). The formation of highly concentrated urine in the dehydrated state is an essential prerequisite for crystal precipitation and deposition. Four of our cases presented with decreased urine output, indicating volume depletion to some extent. Notably, RASIs and/or NSAIDs were concurrently used in most of our patients at the onset of the disease. These drugs might enhance renal hypoperfusion at the glomerular level under the condition of volume depletion, contributing further to the urine concentration. Furthermore, *in vitro* studies showed that prostaglandins may decrease the adhesion ability of calcium oxalate to renal epithelial cells (25, 26). As NSAIDs downregulate prostaglandin production (27), it is feasible to speculate that NSAID use is predisposed to a greater likelihood of adhesion of oxalate crystals to the tubular epithelium. In this regard, we hypothesize that factors, which either decrease urinary flow in renal tubules or increase the adhesion ability of oxalate crystals to the epithelium, contribute convergently to the development of acute oxalate nephropathy when superimposed on hyperoxaluria in DM.

The reported outcomes of AON cases vary in the literature. Some patients experienced complete recovery of kidney function, while the other patients had residual kidney dysfunction with some remaining dialysis dependent (2). The recommended treatments include hydration, oral calcium supplements, alkalization, and correction of hyperoxaluria-enabling conditions. We generally followed these treatment strategies after AON diagnosis was established in our center. The role of alkalization is controversial, as oxalate is pH independent. Previous studies have reported inconsistent impacts on the recovery of kidney function by oral citrate supplements and intravenous fluids containing sodium bicarbonate (8, 28). We provided citrate supplementation not only for alkalization, but also to improve the solubility of oxalate. Many nephrologists also empirically use steroids for the management of AON, without confirming the effectiveness of steroids to date. We also administered a course of corticosteroid treatment to 4 patients with severely elevated serum creatinine levels in the present study. They were followed in the outpatient unit after discharge and instructed to taper corticosteroids gradually to a full stop. Although all the 4 patients showed improvement in kidney function to different degrees at the end of follow-up, 2 of them remained with kidney dysfunction. Of the two patients who did not receive corticosteroid treatment, one patient had eGFR recovered to over 90 ml/min/1.73 m^2^, while the other patient remained with kidney dysfunction. The recovery rate of kidney function was equal between patients with and without corticosteroid treatment. Given the small number of cases in the present study, it is hard for us to judge the effectiveness of corticosteroid treatment. A better understanding of the pathophysiology of oxalate nephropathy might be helpful to establish a more precise treatment for oxalate nephropathy. The inflammasome has been identified to play a critical role in the process of oxalate-induced epithelial injury in recent studies (4). Interleukin-1β (IL-1β) is released upon activation of the NLRP3 inflammasome (4, 29). Administration of an IL-1 receptor antagonist (IL-1ra) has been demonstrated to attenuate oxalate-induced AKI in an animal model (4). Whether IL-1ra treatment is effective in the treatment of AON in clinical practice needs to be studied further.

The strengths of our study are the comprehensive evaluation of the clinical and pathological features, natural history, and prognosis of a series of diabetic patients with biopsy-proven acute oxalate nephropathy. However, some important limitations should be noted. First, the study has inherent limitations of retrospective studies with limited cases, which weaken the strength of making “cause and effect” conclusions. Second, we did not perform special testing with labeled oxalate with urinary recovery to definitely rule out enteric sources at the time of diagnosis, which could not be supplemented given the retrospective nature of the study, and we did not examine blood glyoxalate or glyoxal levels. Third, we did not have data on 24-h urinary excretion of oxalate. We examine spot urine oxalate levels adjusted by urine creatinine, without significantly elevated levels found. One possibility is that the urine sample was collected several days after disease onset. Urinary oxalate excretion might have declined from the initial high level due to the alleviation of kidney dysfunction. In addition, no definite threshold of urinary oxalate concentration is established above which deposition in kidneys is inevitable. Coexisting factors might also play important roles in the process of deposition in addition to urinary oxalate excretion *per se*.

## Conclusion

Acute oxalate nephropathy is a rare but potentially devastating trigger of AKI in patients with DM. Physicians should be more alert about this condition, especially in the setting of oxalate precipitation/attachment-enabling conditions, and perform the renal biopsy in time to establish the diagnosis. How to properly treat patients to alleviate oxalate-induced injury needs to be studied further.

## Data availability statement

The original contributions presented in this study are included in the article/Supplementary material, further inquiries can be directed to the corresponding author.

## Ethics statement

The studies involving human participants were reviewed and approved by Peking University First Hospital, approval number: 2017[1333]. The patients/participants provided their written informed consent to participate in this study. Written informed consent was obtained from the individual(s) for the publication of any potentially identifiable images or data included in this article.

## Author contributions

DB and YW analyzed and interpreted the patient data and were major contributors to writing the manuscript. XY performed interpretation of pathological data. MZ interpreted the clinical data and substantively revised it. All authors have contributed to the article and approved the submitted version of the manuscript.

## Supplementary material

Supplementary Figure 1 Polarized light microscopy image **(A)** and PAS staining **(B)** of the renal biopsy of the six patients with acute oxalate nephropathy. Original magnification 200X **(A)** and 100X **(B)**.
