# Supplementary figures and images for: Acute oxalate nephropathy: A potential cause of acute kidney injury in diabetes mellitus—A case series from a single center
Source: Front Med (Lausanne). 2022 Aug 26;9:929880. doi: 10.3389/fmed.2022.929880 (PMC9484473; doi:10.3389/fmed.2022.929880)

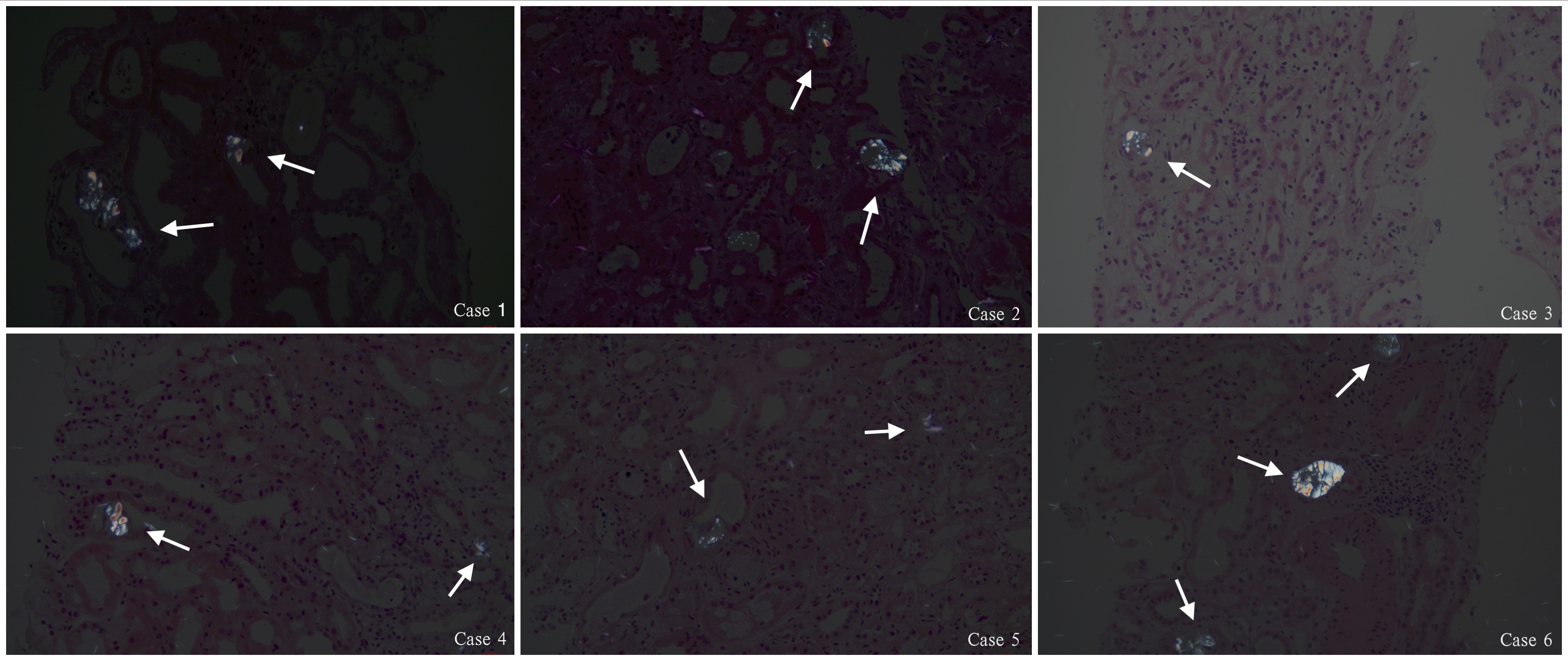

Supplement: Supplementary Figure 1 — Polarized light microscopy image (A) and PAS staining (B) of the renal biopsy of the six patients with acute oxalate nephropathy. Original magnification 200X (A) and 100X (B). [file Image_1.jpeg]

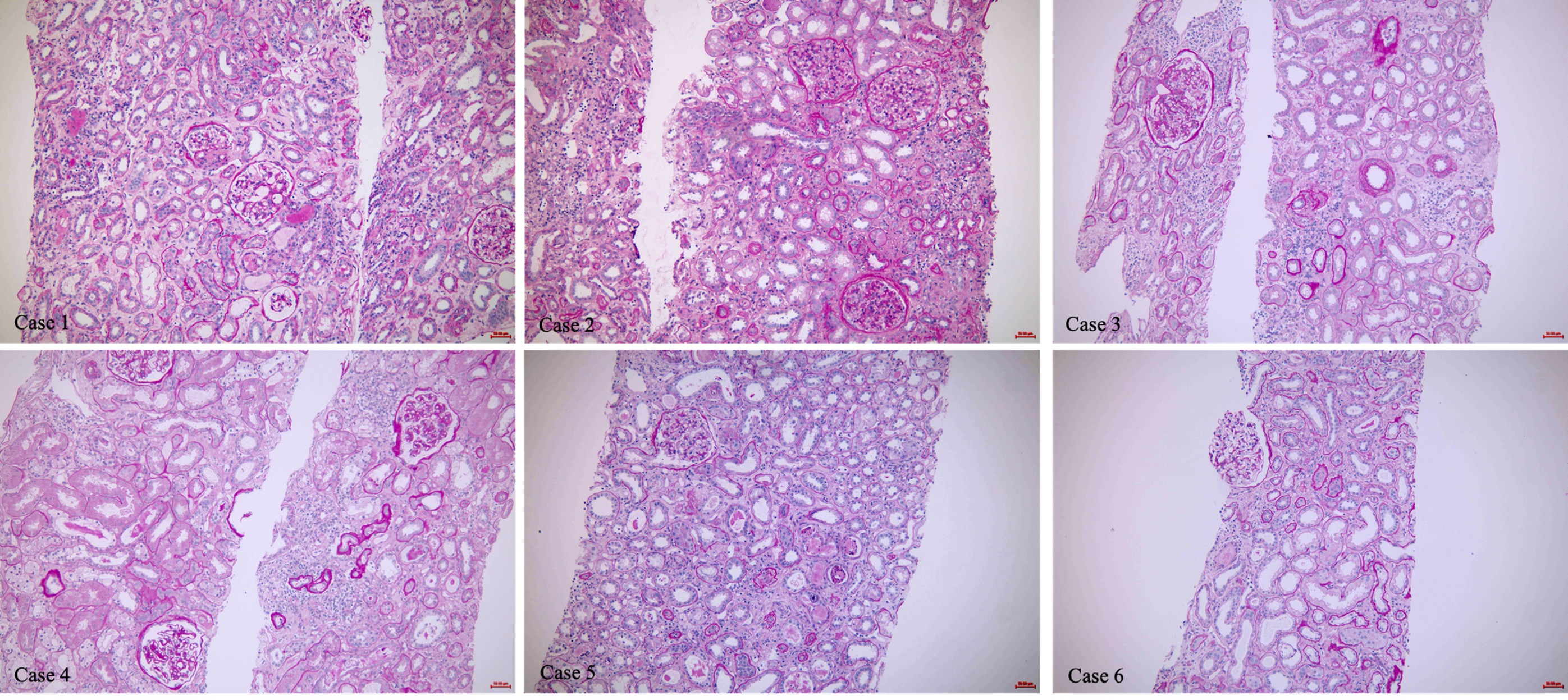

Supplement: Supplementary file 2 [file Image_2.jpeg]
